# Why gone too soon? Examining social determinants of neonatal deaths in northwest Ethiopia using the three delay model approach

**DOI:** 10.1186/s12887-017-0967-9

**Published:** 2017-12-28

**Authors:** Tariku Nigatu Bogale, Abebaw Gebeyehu Worku, Gashaw Andargie Bikis, Zemene Tigabu Kebede

**Affiliations:** 1 Institute of Public Health, University of Gondar, Gondar, Ethiopia; 2 Amhara Regional Health Bureau, Bahir dar, Ethiopia

**Keywords:** Social autopsy, Delays in care seeking, Neonatal mortality

## Abstract

**Background:**

Without improving the survival of newborns, meaningful reduction in under-five mortality is difficult. Most neonatal deaths are preventable when appropriate and timely care is sought. In Ethiopia, there is lack of evidence on the type and contribution of delays in treatment seeking to neonatal deaths.

**Methods:**

A community based social autopsy (SA) of 39 neonatal deaths was conducted from March 16 to 24, 2016 in Dabat Health and Demographic Surveillance System (HDSS) in northwest Ethiopia. The result was linked with verbal autopsy (VA) information completed for each of the deaths as part of the ongoing HDSS. The SA tool was adapted from INDEPTH Network. Three delay model approach was used to classify the delay types that contributed for the deaths investigated. Descriptive statistics was used to analyze the data.

**Results:**

SA was completed for 37 (94.9%) of the 39 neonatal deaths. Of all the deaths, 51.3% (19/37) of them occurred within the first 24 h, 75.6% (28/37) within the first 6 days and the remaining in 7–28 days. Birth asphyxia was the leading cause of death (34%) followed by bacterial sepsis (31%) and prematurity (16%). The median time from recognition of illness to initiation of modern treatment was 1 day (IQR 1–2.5 days). Delay in treatment seeking outside home (delay one) was associated with 81% of the deaths. Delay in receiving care at a health facility (delay three) and delay in transport (delay two) were associated with 16 and 3% of the deaths, respectively. The major contributors of death for delay one were bacterial sepsis (33.3%), birth asphyxia (30%), unspecified illness (20%) and acute lower respiratory tract illnesses (6.7%). For delay three, the major causes of death included birth asphyxia (50%), prematurity (33.3%) and bacterial sepsis (16.7%).

**Conclusions:**

Delays created at home and at health facility were the major delays contributing to the death of newborns. More focus has to be given in improving delays at home and at health facility.

## Background

Without improving the survival of newborns, meaningful impact on child survival would not be possible [1]. This is because globally neonatal mortality contributes nearly half (45%) of the under-five deaths and is estimated to grow by more than half (52%) by 2030 [2]. The increasing trend in the contribution of neonatal mortality is attributed to the slow decline in neonatal mortality compared to post-neonatal under-five deaths [3–5]. In Ethiopia, neonatal mortality reduction is inadequate [6]. The contribution of neonatal mortality to the overall under-five mortality is increasing from time to time reaching 44% in 2013 [7].

The first 28 days of life is very difficult time. Particularly, the rate of death during the first 1 week of life is higher than any other time in life [8]. Globally, two-third of neonatal deaths occur in just 10 countries [5]. The vast majority of these deaths happen in resource limited settings [8, 9]. Linked with the high level of domiciliary delivery in developing countries, most deaths occur at home [8].

Most of the deaths in newborns are preventable when appropriate and timely care is sought [10]. However, despite evidences of correlation between treatment seeking at health facility and neonatal mortality [11–13], some communities still accept newborn deaths as inevitable and don’t demand medical care [14]. In some cultures, restriction of newborns and mothers at home with reasons like uncleanliness and fear of malevolent spirits delay health and treatment seeking behavior [15].

Studies in developing countries demonstrated the application of the three delay model approach to investigate the contribution of delays in care seeking to neonatal and perinatal deaths [16–18]. These studies were based on the work of Thaddeus and Maine (1994) that developed the three delay model to understand the social determinants of maternal deaths [19]. The model included; Delays in recognizing problems and deciding to seek care (delay one), Delays in transportation to reach appropriate care (delay two) and Delays in receiving appropriate care at the health facility (delay three).

Because of lack of civil registration system and unreliability of facility based death records, some developing countries rely on verbal autopsy (VA) to estimate cause specific mortality [8, 20, 21]. However, the VA does not assess care seeking by caretakers before death. This limitation is overcome by social autopsy (SA). Both SA and VA use interview questions with caretakers of the deceased to illicit the required information about the death being investigated.

Recently, the use of SA is being advocated to improve neonatal and child health programs in low-income countries [22]. This is because understanding of the causes of the deaths is critical for prevention. Countries that don’t know the reason why people die cannot realize the full potential of their health system [23]. Thus, the SA provides useful information concerning any modifiable factor at home, in the community and at health facility and referral mechanisms for policy, planning, monitoring and evaluation.

In Ethiopia, there is lack of evidence concerning the contribution of delays in treatment seeking to neonatal deaths. This study aims to investigate the delays in care seeking that are associated with newborn deaths in northwest Ethiopia using the three-delay model approach.

## Methods

### Study area and setting

The study was conducted at Dabat Health and Demographic Surveillance System (HDSS) located in Dabat District in northwest Ethiopia. The HDSS is located around 760 km from Addis Ababa and 75 km from Gondar town. The district has an estimated population of 145,458 individuals living in 27 rural and 3 urban kebeles (the smallest administrative unit). The livelihood of the area is mainly subsistence farming. The district has six health centers and 29 health posts, besides private clinics and drug stores, providing health services to the community. The HDSS covers 13 randomly selected kebeles (four urban and nine rural kebeles) in different ecological zones (high land, middle land, and low land). The site has been established by the University of Gondar and became operational since November 1996. The site collects longitudinal data on vital events like births, deaths, migration, pregnancy and its outcomes [24].

### Study design and period

Community based SA was conducted from March 16 to 24, 2016. The SA included all neonatal deaths in the HDSS that occurred in the past 18 months prior to the survey. Information on household contact details and causes of death was collected from the completed VA from the HDSS record.

### Study population, sample size and sampling technique

Each death in the first 28 days of life is recorded as part of an ongoing HDSS. For each death, the HDSS completes VA after allowing 4 weeks of mourning period to confirm the cause of death (COD). Our study identified 64 deaths that occurred from October 2013 to September 2015 from the HDSS. Twenty-five of these deaths were stillbirths and were excluded from our study. Then, we conducted SA for the remaining 39 neonatal deaths identified by VA. We contacted primary caretakers (mothers, fathers, grandparents or siblings) interviewed during the VA to complete the SA tool. If the primary caretaker was not available during the first visit, a second visit was made. If still unavailable, the most knowledgeable person in the household who knew about the death of the indexed newborn was interviewed. The information we collected from the SA was eventually linked with the COD data obtained from the VA from the HDSS.

### Data collection

A modified SA tool was used to identify social determinant of newborn deaths. The tool was adapted from INDEPTH Network (http://www.indepth-network.org/) and has open and closed ended questions. In the open-ended questions, caretakers were asked to narrate about the indexed newborn death. In the closed ended questions, caretakers were asked information on the kind of treatment and health services they used before the death of the newborn. The closed ended questions were slightly modified to reflect the local contexts.

The HDSS’ supervisors and data collectors who know the area very well and have several years of experience were used for data collection. Utilizing data collectors and supervisors having such experience and knowledge about the area and households contribute to data quality.

### Ascertainment of cause of death (COD) and delay type

A panel of physicians used the INDEPTH Standardized Verbal Autopsy questionnaire, (http://www.indepth-network.org/), to determine the COD and type of death (stillbirth versus neonatal death). Two trained physicians independently reviewed the completed VA tools. In case of disagreement between the review outcomes of the two physicians, a third coder (a trained physician), blinded about the review outcomes of the first two coders, provided further independent assessment. When two of the three coders agreed, the diagnosis was taken as final. The COD determined in this way is *‘the probable cause of death’*. If no agreement among the three independent coders on the COD, the case was classified as unknown in accordance with the International Classification of Diseases, 10th Revision (ICD) [25].

The determination of the delay types was made in similar manner. The first two authors of this paper, TN and AG, independently reviewed each deaths for the type of delay using indicators adapted from a study by INDEPTH Network (Table 1) [26]. In case of disagreement between TN and AG, the third researcher, ZT, reviewed the deaths. When two of the three agreed, the decision was taken as final. Where there was disagreement among the three assessors, they discussed together and reached consensus.

**Table 1:** Indicators adapted from INDEPTH study used for classifying the delay types

1. Indicators of delay one: Home delay	
1.1. Newborns whose caregivers did not mention at least one danger sign	
1.2. Newborns with possibly severe or severe symptom who were treated at home	
1.3. Newborns only receiving treatment at home without going outside for care	
1.4. Newborns with severe symptoms who were brought outside the home for care after a day	
1.5. Newborns who only received informal health care for their fatal illnesses as both first and last source of care	
1.6. Newborns not going for referral because of caretakers decision making	
1.7. Caretakers did not take action at home or outside of home for different reasons^a^	
2. Indicators for delay two: Transport delay	
1.8. Delaying >2 h to reach first or last provider	
1.9. Caretakers not going for referral because of lack of money for transport	
3. Indicators of delay three: Facility level delay	
1.10. Newborns obtaining treatment from providers after >1 h from first or last provider	
1.11. Newborns referred because of lack of equipment or lack of drugs	
1.12. Newborns who did not receive any treatment after visiting first or last formal provider	

^a^indicator included in the list by the researchers

### Data analysis considerations

Data from each completed SA and from the corresponding VA were entered in to EpiData software version 3.1. The EpiData database was exported to statistical package for social sciences (SPSS) version 20 for analysis. After cleaning, descriptive analysis was conducted for the data. Percentages, means and medians were used to summarize the data. Variability was assessed with measures of dispersion such as standard deviation and interquartile range (IQR) as appropriate. Households’ socio-economic position was categorized in to quintiles: poorest, poor, average, rich and richest. The index was constructed using household asset and characteristics data using the principal component analysis. The qualitative part of the SA questionnaire was used to provide information to help identify the delay types in treatment seeking that contributed to the deaths.

### Ethical considerations

Ethical clearance was obtained from the Institutional Review Board (IRB) of the University of Gondar, Ethiopia. Permissions were obtained from Dabat HDSS site to use the VA data and from Dabat woreda administration and the kebeles the SA was conducted. Verbal informed consent was obtained from the VA and the SA respondents. This method of data collection was approved by the IRB of Gondar University.

## Results

Social Autopsy was conducted for 37 (94.9%) of the 39 deaths. The families for two of the neonatal deaths had left the study area by the time the SA was conducted. Of all the deaths, 51.3% (19/37), 75.6% (28/37) and 24.3% (9/37) occurred in the first 24 h, 0–6 days and between 7 and 28 days of life, respectively. Two third of the mothers, 67.4 (25/37), received at least one antenatal care during pregnancy. However, only a little more than a quarter, 27% (10/37), of the deliveries took place at a health facility. More than half (56.8% (21/37)) of the mothers were tested for HIV to prevent mother to child transmission of HIV. Nearly a third, 32.4% (12/37), of the deceased newborns were breastfed of which 33.3% (4/12) were given breast milk within 1 h. Of those who were born at home or on the way to health facility, 40.7%(11/27), cut the cord using used razor blades. The umbilical cord was not tied for most, 88.9% (24/27), of the neonates born at home (Table 2).

**Table 2:** Socio-economic status and maternal health seeking behavior during pregnancy and immediate postpartum, northwest Ethiopia, March 2016

Variable	Number	Percent
Socio-economic position		
Poorest	7	18.9
Poor	8	21.6
Average	7	18.9
Rich	8	21.6
Richest	7	18.9
Time to death		
Within 24 h	19	51.4
Within 6 days	9	24.3
Between 7 and 28 days	9	24.3
ANC attendance		
No	12	32.4
Yes	25	67.6
Four or more ANC		
No	16	64
Yes	9	36
Place of delivery		
Health facility	10	27.0
Home	25	67.6
On the way to health facility	2	5.4
HIV test during pregnancy		
No	13	35.1
Yes	21	56.8
Didn’t remember	3	8.1
Iron folic acid intake during pregnancy		
No	18	48.6
Yes	18	48.6
Didn’t remember	1	2.7
Newborn was breastfed		
No	24	64.9
Yes	12	32.4
Did not remember	1	2.7
Time breast feeding started		
Within 1 h	4	33.3%
After 1 h	8	66.7
Material used to cut the cord for deliveries at home or on the way to health facility		
New razor blade	13	48.1
Used razor blade	11	40.7
Scissors	2	7.4
I don’t remember	1	3.7
Umbilical cord was tied		
No	24	88.9
Yes	3	11.1

### Causes of death (COD)

**Fig. 1:**
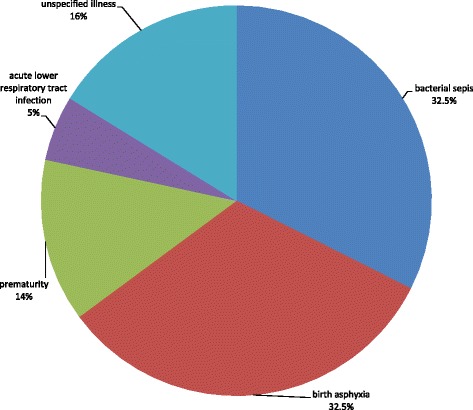
Causes of neonatal deaths, northwest Ethiopia, March 2016

Birth asphyxia and bacterial sepsis were the leading causes of death contributing for 32.5% (12/37) of the deaths, followed by prematurity, which contributed 14% (5/37) (Fig. 1).

The major cause of death in the first 24 h was birth asphyxia, 52.6% (10/19), followed by unspecified illnesses, 21.1%(4/19), and prematurity, 15.8% (3/19). For neonatal deaths between 1 and 6 and 7–28 days, the major COD was bacterial sepsis with increasing contribution, 44.4%(4/9), and 66.7% (6/9), respectively (Table 3).

**Table 3:** Causes of newborn death by time from birth to death, northwest Ethiopia, March 2016

Cause of death	Time from birth to death					
	Within 24 h		1–6 days		7–28 days	
	No	%	No	%	No	%
Bacterial sepsis	2	10.5	4	44.4	6	66.7
Birth asphyxia	10	52.6	2	22.2	0	0
Prematurity	3	15.8	1	11.1	1	11.1
Acute lower respiratory tract infection	0	0	0	0	2	22.2
Unspecified	4	21.1	2	22.2	0	0
Total	19	100	9	100	9	100

Perceived causes of the deaths reported by caretakers included; fast breathing, excessive cord bleeding, cord tie around the neck of newborn during delivery, born too early to survive, intake of medications for treatment of illnesses during the indexed pregnancy, exposure of the newborn and the mother to sunlight and bewitchment or evil eyes.

### Delays contributing to newborn deaths

**Fig. 2:**
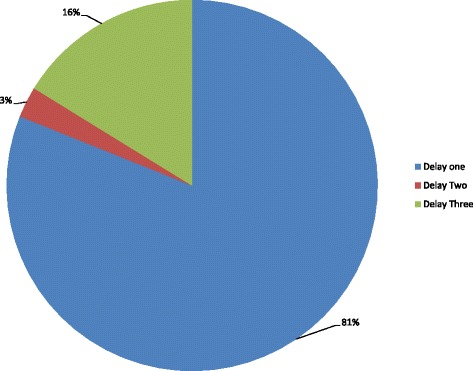
Contributing delays to neonatal deaths, northwest Ethiopia, Mach 2016

All of the deaths included in this study were associated to one of the three delay types. Delay in treatment seeking outside home (delay one) was associated with 81% (30/37) of the deaths (Fig. 2).

Of all the deaths investigated, 70.31% (26/37) of them did not take any treatment. The median time from recognition of illness to modern treatment seeking was 1 day (IQR 1–2.5 days). Similarly, the median time from recognition of illness to death of the newborn was 10 h (IQR 0.5–72 h). The major delays associated with early and late neonatal deaths were delay one followed by delay three (Table 4).

**Table 4:** Delays associated with neonatal deaths at different time after births of the indexed newborns, northwest Ethiopia, March 2016

Type of Delay	Time of death					
	Within 24 h		1–6 days		7–28 days	
	No	%	No	%	No	%
Delay I	17	89.5	6	66.7	7	77.8
Delay II	0	0	0	0	1	11.1
Delay III	2	10.5	3	33.3	1	11.1
Total	19	100	9	100	9	100

The commonest reasons given for not taking newborns for treatment included; newborn was ‘*only blood’* and not old enough to receive treatment, belief that the mother and the newborn shouldn’t go out of home before baptism, illness onset was at night and abrupt, hence, could not get time to take newborn for treatment, and expectation of self-recovery from illness.

Delay in receiving care at a health facility (delay three) was the second largest delay associated with 16% (6/37) of the deaths (Fig. 2). Of the 10 (27%) caretakers who sought modern treatment for their sick newborns, 7(70%) of them went to hospitals and the remaining visited health centers. Delay in transport (delay two) was associated with 3% (1/37) of the deaths.

The major contributors of death for delay one were bacterial sepsis (33.3%), birth asphyxia (30%), unspecified illnesses (20%) and acute lower respiratory tract illnesses (6.7%). Bacterial sepsis was associated with all the death for delay two. For delay three, the major cause of death included birth asphyxia (50%), followed by prematurity (33.3%) and bacterial sepsis (16.7%).

Nearly a third of the newborns, 30% (3/10), delivered at health facilities and more than half, 56% (14/25), of the newborns delivered at home died within the first 24 h (Table 5). The three deaths in the first 24 h among newborns delivered at health facilities occurred within the health facilities. Referral due to lack of equipment and drugs was mentioned as the reason for the deaths. The remaining seven deaths occurred after discharge. Of all the newborns delivered at health facilities, 60% (6/10) and 40% (4/10) of them were associated with delay type three and one, respectively. Similarly, 96% (24/25) of the deaths among newborns delivered at home were associated with delay type one.

**Table 5:** Time neonatal death by place of delivery northwest Ethiopia, March 2016

Place of Delivery	0–24 h		1–6 days		7–28 days		Total
	No	%	No	%	No	%	
Health facility^a^	3	30	5	50	2	20	10
Home	14	56	4	16	7	28	25
On the way to health facility	2	100	0	0	0	2	2
Total	19		9		9		37

^a^health center and hospital

## Discussion

This study showed that more than half of the neonatal deaths occurred in the first 24 h. The number of deaths in the early neonatal period was more than three times the number of deaths in the late neonatal period. This was more or less similar to the national estimate of early and late neonatal mortality in Ethiopia which stood at 79 and 21%, respectively [27].

Two third of the mothers accessed at least one antenatal care during pregnancy. However, less than a third of the neonates were delivered at health facility. This suggests that non-adherence and low uptake of skilled maternity services could be contributing to the deaths.

Birth asphyxia, bacterial sepsis and prematurity were associated with 81% of the neonatal deaths. The highest number of early and late neonatal deaths was caused by birth asphyxia and bacterial sepsis, respectively. The three leading causes of neonatal deaths in this study were also reported to be associated with deaths of newborns in a facility based study in Namibia [28]. However, their relative contributions to the deaths were different in the two studies. In this study, birth asphyxia and bacterial sepsis were the leading causes, where as in the Namibian study, the leading cause was prematurity. The difference could be attributed to the difference in the study populations of the two studies.

Even though some caretakers reported medically recognized danger signs as causes for the death of newborns, still differences exist between what was perceived locally and known medically about the causes. A study in central and southern Ethiopia also reported discordance between locally known danger signs and medically recognized dangers signs among caretakers [29].

Not all neonatal deaths are preventable. Despite presence or absence of delay, some neonatal deaths are unavoidable [30]. However, in this study, all of the causes of the neonatal deaths were preventable with early and timely care of newborns. Despite disagreements among coders, the independent assessment results of deaths coded as unspecified were also preventable. Hence, all of the deaths were assigned one or the other delay type based on the criteria. Accordingly, majority of the neonatal deaths in our study were associated with delays in treatment seeking outside home (delay one). This was followed by the delay in initiating treatment at health facility (delay three). The two types of delays (delay one and three) were associated with 97% of the deaths. The fact that delay one was the major contributor to neonatal deaths in this study could be partly explained by the belief in postpartum home restriction of newborns and mothers before baptism. Similar home restriction of mothers and newborns was reported in a nationwide study in Ethiopia [31]. Even though the restriction allow a period of rest, repair and breastfeeding, it is detrimental to treatment seeking when either the mother or the newborn is very sick.

Despite differences in the percentage contribution of delay one and three to neonatal deaths, the same delay types were also incriminated to have contributed to the deaths of newborns in a study in Uganda [16]. Difference in socioeconomics, demographics, the health care system and cultural practices could be the reason for the difference in the two studies.

The best way to prevent newborn deaths is to ensure that essential care is provided around labor, delivery and the immediate postpartum period [32]. Lack of appropriate care during this period results in significant ill health and even death [33]. In this study, more than a quarter, 27% (10/37), of the newborns who died had access to facility delivery. Particularly, nearly a third of the newborns died within the health facilities where they were born. This calls for an urgent look into the quality of services in our health facilities.

### Limitation of the study

The results of this study need to be interpreted in light of its limitations. Due to the small size of the sample and the cultural diversity of the population in Ethiopia, the result of this study may not be generalizable to the entire country. However, care has been made to get as accurate information as possible for the deaths reviewed. The type and magnitude of delays associated with the neonatal deaths can also be affected by misclassification bias that might be introduced when trying to differentiate neonatal deaths from stillbirths during the VA. However, since the VA was done according to the standard, the limitation is thought to be minimal.

A long recall period that extends as far as 18 months introduces recall bias. The fact that most of the respondents were primary caregivers of the deceased newborns also introduces social desirability bias as they may not accurately report actions they took during illness. Changes in the person interviewed between the time the VA and SA were administered might affect the responses as well.

## Conclusion

The major delays in treatment seeking contributing to the death of newborns were delays created at home and at health facility. Therefore, interventions must focus on avoiding those delays at home and at health facility. To this effect, strengthening the existing community networks and the health extension program is very important. Improving skilled delivery uptake, recognition of danger signs and working with religious leaders help improve newborn survival in the area.

## Abbreviations

ANC: Antenatal care
COD: Cause of death
HDSS: Health and demographic surveillance system
HIV: Human immunodeficiency virus
ICD: International classification of diseases
IQR: Interquartile range
IRB: Institutional review board
SA: Social autopsy
SPSS: Statistical package for social sciences
VA: Verbal autopsy
